# Development and Evaluation of an iPad App for Measuring the Cost of a Nutritious Diet

**DOI:** 10.2196/mhealth.3314

**Published:** 2014-12-04

**Authors:** Claire Palermo, Dharani Perera-Schulz, Anitha Kannan, Helen Truby, Alan Shiell, Sindhu Emilda, Steve Quenette

**Affiliations:** ^1^Department of Nutrition and DieteticsMonash UniversityNotting HillAustralia; ^2^Monash University E SolutionsClaytonAustralia; ^3^Monash University E-Research CentreClaytonAustralia; ^4^Centre for Excellence in Intervention and Prevention ScienceCarltonAustralia

**Keywords:** portable digital device, iPad, healthy food, food cost

## Abstract

**Background:**

Monitoring food costs informs governments of the affordability of healthy diets. Many countries have adopted a standardized healthy food basket. The Victorian Healthy Food Basket contains 44 food items necessary to meet the nutritional requirements of four different Australian family types for a fortnight.

**Objective:**

The aim of this study was to describe the development of a new iPad app as core to the implementation of the Victorian Healthy Food Basket. The app significantly automates the data collection. We evaluate if the new technology enhanced the quality and efficacy of the research.

**Methods:**

Time taken for data collection and entry was recorded. Semi-structured evaluative interviews were conducted with five field workers during the pilot of the iPad app. Field workers were familiar with previous manual data collection methods. Qualitative process evaluation data was summarized against key evaluation questions.

**Results:**

Field workers reported that using the iPad for data collection resulted in increased data accuracy, time savings, and efficient data management, and was preferred over manual collection.

**Conclusions:**

Portable digital devices may be considered to improve and extend data collection in the field of food cost monitoring.

## Introduction

The cost of nutritious food is a key factor influencing food access, choice, and therefore impacts on health [1]. Socially and economically disadvantaged populations have greater rates of nutrition related diseases. Monitoring the cost and availability of a nutritious basket of food can provide useful information to assess both economic and physical access to nutritious food in a region, as well as changes in affordability over time, and is vital information to assist in informing policy. Healthy food baskets are a tool implemented regionally to reliably achieve this. A range of healthy food baskets have been developed internationally to assess and monitor the cost of an ideal nutritious diet [2-5]. Healthy food baskets have been deemed to be an effective instrument to assess economic food access [6]. The Victorian Healthy Food Basket (VHFB) is a shopping list of the specific quantities of 44 commonly consumed foods that, as a whole, meet the nutrient requirements of four reference families for a fortnight [7]. Checking the price of these items at supermarkets makes it possible to calculate the cost of a nutritious diet, monitor changes over time, and compare the cost of healthy diet to income data to assess affordability. In Australia the distance between a store and the nearest capital city can be over 2000 km [3], creating a need for efficient mechanisms for the collection of data. We focus on a study in the state of Victoria, Australia, covering 115 stores over 27 geographically and demographically distinct areas with a total population of 2,525,534.

Practical implementations of healthy food baskets rely significantly on data collection. Traditional approaches to collecting data on the cost of items in a healthy food basket involve simple, manual data collection techniques, including pen and paper at the source of observation. This data is then entered into computerized spread sheets for the purpose of data analysis. This form of data collection and management can be inaccurate, labor-intensive, and therefore inefficient [8-11]. Newer technology developments such as portable digital devices (Personal Digital Assistant [PDA] and smart touch devices) and wireless technologies have opened many opportunities for the way we collect, store, manage, and share survey data. These advances are making data collection and sharing faster, easier, and more accurate [8-11].

**Figure 1:**
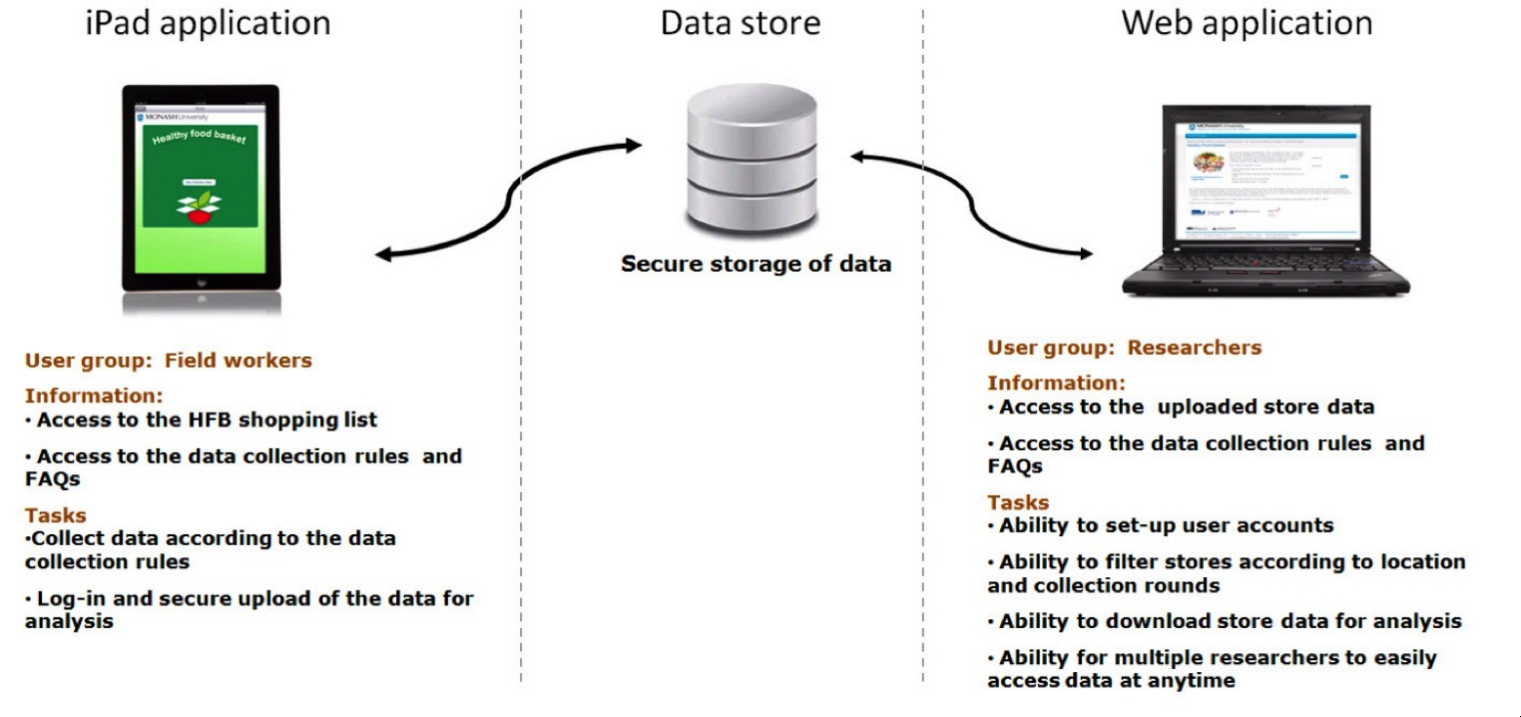
Diagrammatic representation of the technological solution.

**Figure 2:**
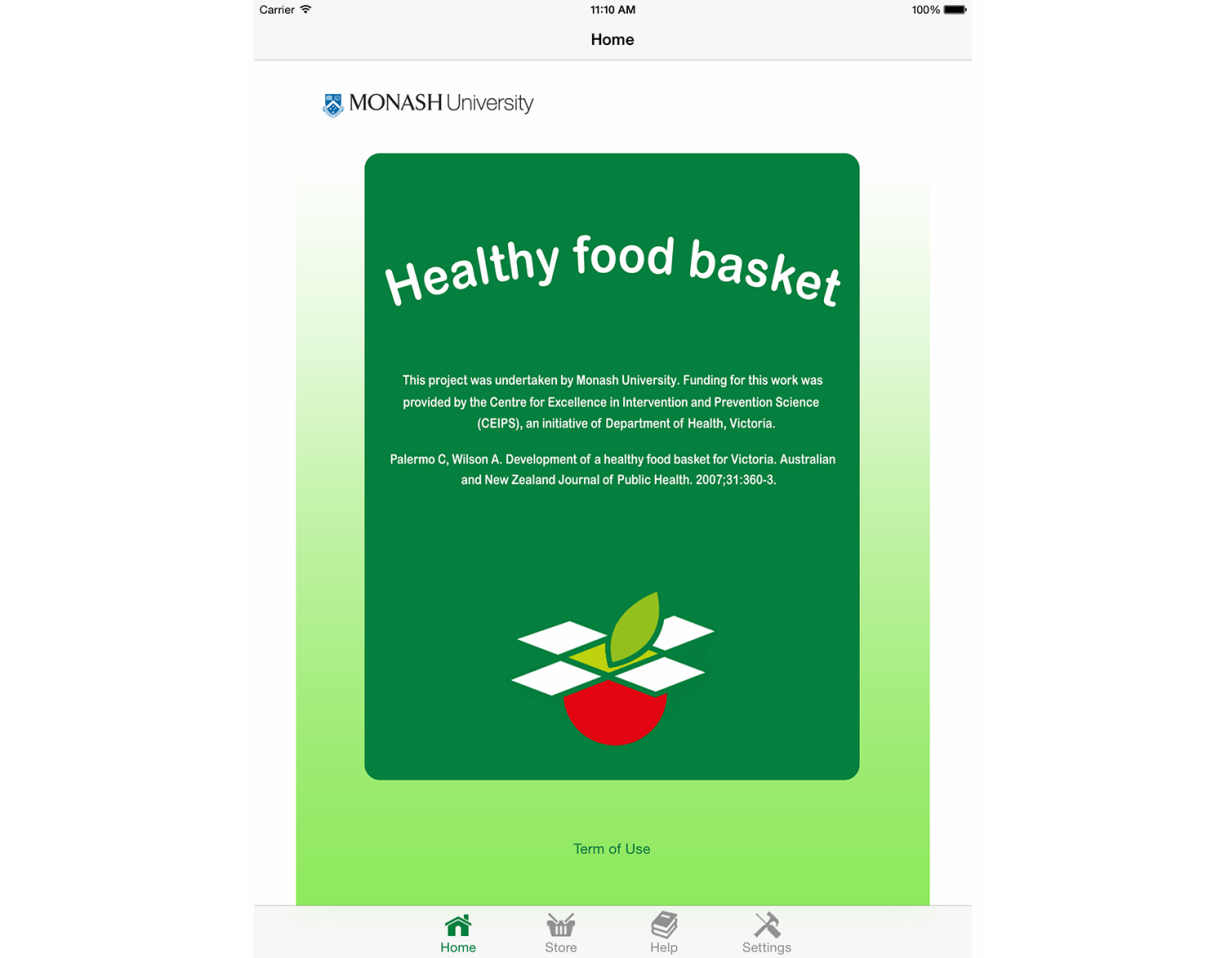
Home screen healthy food basket iPad app.

An iPad app aimed to enhance the quality and efficacy of the research and was complemented by a Java Server app that provides a data management and analysis platform (Figure 1). The Java Server app is built using Model View Controller (MVC) and Inversion of Control (IoC) design patterns that employ Struts2 technology to generate interactive webpage content, with persistence to a database achieved using Hibernate JPA. The app is currently deployed on an Apache Tomcat server using a PostgreSQL database. The iPad app was developed to incorporate instructions for data collection as well as data entry fields with quality controls (Figure 2). Upon completion of the data entry, the field workers upload the shopping lists to the central data store, eliminating the need for manual data entry. The app was implemented with the ability to seamlessly manage data capture and data upload even when connectivity to the mobile network is not available. The iPad app had basic error validation and prevention mechanisms such as a format for price, quantity, and address fields. Error messages were displayed if users entered data that did not match the agreed formats, drop-down boxes so that users were limited to a set of valid data options, and if users were not able to upload prices/data from stores that did not have a completed data set. The app was freely available from the Apple App Store with conditions for use for research purpose highlighted as a condition of the download. While anyone is able to download the app, only those trained in rule for data collection and use of the iPad are given user accounts by the research team to upload their data to the central Web-based store. This paper describes the development and process evaluation of the iPad app for the collection of healthy food basket data.

## Methods

The iPad app was piloted to test for user-friendliness. All field workers (n=6) who were selected to pilot the technology were invited to be part of the evaluation. They all had previous experience with the paper-based, manual data collection processes and consented to be interviewed to evaluate the new technology.

A process evaluation was conducted using semi-structured interviews with five field workers (83% response rate) at the completion of the pilot. The focus of the process evaluation was on the quality and efficiency of, and satisfaction with, the technology and perceptions of improvements from previous paper-based methods (Table 1) [11]. The short interview explored the experience of using the iPad app for data collection and entry. Interviews were audio-recorded and notes taken. Interviews were analyzed by one author (DPS) using an approach whereby audio-recording and notes were reviewed, text coded, and grouped into categories against the process evaluation questions [12]. The analysis was verified by another author (CP) through independent review of notes from interviews.

**Table 1:** Interview questions asked from field workers and the focus of inquiry for process evaluation.

Questions of inquiry	Inquiry logic
Approach to data collection using the iPad app	Quality of app, visual appeal and functionality
Strengths of data collection using the iPad app compared to paper-based	Efficiency – ease of use, perceived ease compared to paper-based collection
Challenges with data collection and entry using the iPad app	Satisfaction – overall with app and compared to manual collection
Recommendations for future improvement of iPad app	

## Results

### Accuracy

Field workers commented that the iPad app resulted in more accurate data collection with fewer discrepancies. Collecting data in a paper format at the store and entering the data later into an excel file was tedious, error prone, and time-consuming.

When I used to enter data …. I would have about 15-20 stores piled up and start entering them one after the other and you could easily miss a decimal point or an item. But with the iPad … I actually made less mistakes when entering prices… [Field worker number 4].

### Efficiency

The field workers reported that another significant benefit of portable digital device was the time savings made. Compared to the previous paper-based model, field workers found the ability to upload the data directly from the app saved time. Compared to paper-based data collection, using the iPad app was reported to be a fast, easy, and convenient way to collect data whilst in the supermarkets.

I would have to go home and then enter data… Sometimes I had to wait until the file is shared with me. With the iPad, once I have collected the data in the store the work is done. It saves a lot of time.

Field worker number 1

### Ease

The field workers commented that the iPad app was easy to use and they were able to be familiar with the available functionality quickly and with minimal training. Participants commented that having access to the data collection rules in the iPad reduced the need to manage multiple sheets of paper. Field workers recorded that the time taken to collect and upload data was between 45-60 minutes. This varied from previous paper-based collection which took 70-90 minutes in total and was a two-step rather than one-step process. Field workers described entering data using the iPad for the first time took longer than when using pen and paper. However, after the first store the time taken was the same as that of the paper and pen. They explained that this was due to the need to become familiar with the new technology.

I looked at the user guide for about 5 mins and was ready to go. It’s very easy to use.

Field worker 1

We didn’t even look at the guide, the orientation we had was good enough.

Field worker 2

## Discussion

The development of this novel iPad app for the collection of food cost data was perceived to provide a more efficient and accurate form of data collection compared to previous manual-based systems.

Improved data accuracy is a major advantage for using portable digital devices. Previous studies have shown that error validation, prevention, and workflow improvements (such as taking away the need to double handle data entry) in the surveys designed for digital devices significantly reduce the errors seen when data is collected using manual techniques [8,9]. Adding further error prevention and validation mechanisms such as range checks, real-time visibility of missing data, and automatic capture of store addresses using GPS capabilities of the devices are planned for the immediate future. Price scanning using the portable device may also be investigated.

While views of the five field workers in this study are likely to be transferable to others using portable devices for data collection, the small sample size and focus on process evaluation are acknowledged as limitations in the breadth and depth of evaluation data.

There remains a need for a national food and nutrition monitoring system in Australia [14]. The electronic support systems of the healthy food basket could form part of this system. This research provides some evidence to support the idea that using portable digital devices for data collection in the nutrition research area can result in increased data accuracy, time savings, and efficient data management and sharing. The iPad app is available free to use for research purposes (contact corresponding author for more information).

## Abbreviations

IoC: Inversion of Control (IoC)
MVC: Model View Controller
PDA: Portable Digital Assistant
VHFB: Victorian Healthy Food Basket
